# An outbreak of campylobacteriosis at a hotel in England: the ongoing risk due to consumption of chicken liver dishes

**DOI:** 10.1017/S095026882000028X

**Published:** 2020-02-19

**Authors:** A. Wensley, S. Padfield, G. J. Hughes

**Affiliations:** 1 Field Epidemiology, Field Service, National Infection Service, Public Health England, Leeds, UK; 2 Public Health England Yorkshire and Humber, Leeds, UK

**Keywords:** *Campylobacter*, foodborne diseases, gastroenteritis, outbreaks

## Abstract

Despite a sizeable evidence base for the risk of campylobacteriosis associated with eating chicken liver pâté, associated outbreaks continue to occur. In January 2017, six cases of campylobacteriosis reported having eaten a Christmas set-menu meal at the same hotel in North Yorkshire, England on the same day. A retrospective cohort study was undertaken to test the null hypothesis that consumption of individual food items was not associated with an increased risk of illness. There were 19 cases of campylobacteriosis linked to the outbreak; seven confirmed and 12 probable cases. Chicken liver pâté was the food item most strongly associated with illness (*P* < 0.001) with a corresponding high crude relative risk (12.95). This relationship was supported by multivariable analysis, sensitivity analyses and a clear dose–response relationship. Three cases reported an incubation period of <24 h, consistent with other outbreaks of campylobacteriosis associated with consumption of poultry liver. The findings were suggestive of a single point source exposure with a strong association between the consumption of chicken liver pâté and campylobacteriosis. This outbreak highlights that despite evidence that simple cooking techniques can ensure that all campylobacter are killed during cooking, outbreaks continue to occur. Public and professional awareness needs to be raised through a strategic communication plan to reduce the risk of further outbreaks of campylobacteriosis linked to incorrectly cooked chicken liver dishes.

## Introduction

In England and Wales, campylobacteriosis is by far the most commonly reported gastrointestinal infection, with over 56 000 laboratory reports in 2017 [1]. Infection with *Campylobacter* spp. is usually self-limiting with onset of gastroenteritis 2–5 days following exposure [2]. Shorter incubation periods (<1 day) have been reported following consumption of poultry liver [3]. Symptoms may last for several weeks and can lead to long-term post-infection complications such as Guillain–Barré syndrome, reactive arthritis and irritable bowel syndrome [4] and is estimated to have a substantial burden in the United States [5].

The majority of campylobacteriosis cases are termed sporadic, having no established epidemiological link [6]. Where known, in England and worldwide, international travel, consumption of inadequately cooked meat (including chicken), untreated water, unpasteurised milk and handling of raw chicken are the most common risk factors [7 8]. The link between consumption of chicken livers and campylobacteriosis in the United Kingdom (UK) is a long-established one [8–12]. There is strong evidence that pathogens such as *Campylobacter* may exist within internal chicken liver tissues, remaining viable within these tissues even when surfaces of organs have been sterilised [13]. Concern over the risk of campylobacteriosis from chicken liver dishes has been such that a study was funded by the UK Food Standards Agency to develop a protocol and recipe to reliably destroy campylobacters during commercial preparation of chicken liver pâté [14, 15]. In the United States and Australia, blended chicken livers are also recognised as an important risk for campylobacteriosis [13].

In January 2017, a local Environmental Health (EH) team in North Yorkshire, UK was contacted by a member of the public reporting cases of gastroenteritis following a Christmas party event at a hotel on 17 December 2016. The EH team contacted Public Health England (PHE) and an outbreak control team was convened. The hotel is privately run, offering hotel, wedding and conference accommodation as well as a restaurant offering a daily a la carte menu and a Sunday carvery. During the Christmas period, a set menu is offered for groups. Three Christmas parties were held at the hotel on 17 December (47, 10 and 16 attendees, respectively; 73 in total). All three parties ate from the same set menu which included a choice of starter, main course and dessert. Epidemiological and environmental investigations were conducted to determine the source of the outbreak and to implement control measures.

## Methods

### Epidemiological investigation

A retrospective cohort study was used to investigate the cause of the outbreak. The cohort was defined as any persons eating from the Christmas lunch menu at the venue on the 17 December 2016. Local EH staff contacted known attendees from the Christmas parties on 17 December to collect the names of those that visited as part of their groups. Further case finding was undertaken by contacting all confirmed cases of campylobacteriosis resident in the immediate areas after 28 December 2016 to ascertain any links to the venue.

An online questionnaire was distributed either directly or via onward distribution from the organiser of each group to all persons within groups. The questionnaire included questions on symptoms, food and drink items consumed and other potential risk factors for sporadic campylobacteriosis (drinking from a private water supply, drinking unpasteurised milk or products containing unpasteurised milk, close contact with animals or their faeces, participation in outdoor activities, eating poultry or poultry-containing products not served at the hotel on 17 December).

A confirmed case was defined as an individual with a stool sample positive for *Campylobacter* spp. with onset of diarrhoea and/or vomiting between 17 and 22 December 2016 who had eaten at the venue on 17 December 2016. A probable case was defined as an individual with onset of diarrhoea and/or vomiting between 17 and 22 December 2016 who had eaten at the venue on the 17 December 2016 but did not have microbiological confirmation of infection.

### Statistical analysis

Pearson's *χ*^2^ test was used to assess differences in characteristics of cases and non-cases. Risk ratios (RR) with corresponding 95% confidence intervals (CIs) and Fisher's exact *t*-test *P*-values were used to assess crude associations between the consumption of individual food items and illness. Mantel–Haenszel weighted RR (MH_RR_) were calculated for stratified analysis. Multivariable logistic regression was conducted on all exposures with a crude RR > 1 and with an associated *P* < 0.2, age group (0–19, 20–39, 40–59, ≥60 years) and sex. Statistical analysis was undertaken using Stata v15.1 (StataCorp).

### Microbiological and environmental investigation

Cases were identified by culture of *Campylobacter* spp. from faecal specimens submitted via general practitioners and tested according to standard protocols at the local National Health Service microbiology laboratory. As no staff illness was reported, no samples were obtained from this group.

EH staff inspected the hotel premises on 4 January and obtained food samples and environmental swabs on 5 January. All the specimens were sent to the PHE Food Water and Environment (FWE) laboratory for testing for *Campylobacter* spp., *Listeria* spp., coliform bacteria and aerobic colony counts.

## Results

### Descriptive epidemiology

**Fig. 1.**
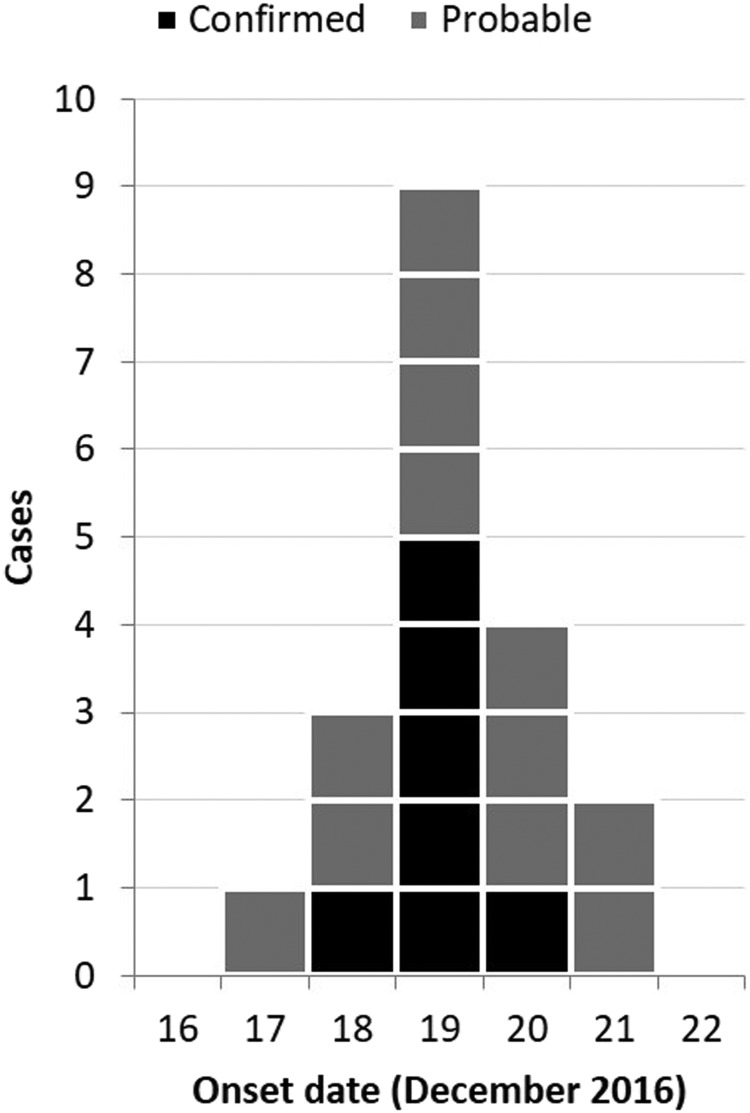
Cases of campylobacteriosis by onset date and case definition during an outbreak of campylobacteriosis at a hotel in England, December 2016 (*n* = 19).

There were 53 completed online questionnaires, of which 19 met one of the case definitions (seven confirmed and 12 probable). No cases linked to the outbreak were found through contacting of routinely ascertained cases in the immediate areas. The median age of the cohort was 45 years (interquartile range 32–52) with no significant difference in age between cases and non-cases (*P* = 0.560). The cohort was predominantly female (*n* = 38, 72%), with no significant difference in the proportion female between cases and non-cases (*P* = 0.112). Cases reported onset of illness between 4 h and 4 days after the meal (Fig. 1). Three cases reported an incubation period of <24 h (4, 13 and 22 h). Almost all cases (18/19, 95%) reported diarrhoea; other symptoms reported included abdominal pain (17/19, 89%), nausea (15/19, 79%), headache (12/19, 63%), fever (11/19, 58%), body aches (11/19, 58%), bloody stools (5/19, 26%) and vomiting (5/19, 26%).

### Analytical epidemiology

#### Single variable and stratified analysis

Chicken liver pâté was the food item most strongly associated with illness in single variable analysis (Table 1) and explained a high percentage (89%, 17/19) of cases. All confirmed cases reported consuming chicken liver pâté. A clear, monotonic dose–response relationship was observed when comparing different reported quantities of chicken liver pâté eaten with not reporting eating any chicken liver pâté: less than a portion (RR = 10.67, 95% CI 2.45–46.42, *P* = 0.002), a whole portion (RR = 13.54, 95% CI 3.42–53.56, *P* < 0.001) and more than a portion (RR = 16.75, 95% CI 4.32–64.94, *P* < 0.001). The only other food item with a crude RR > 1 and that explained >10 cases was consumption of mints (11 cases, relative risk (RR) = 1.94). Other food items with an associated *P* < 0.2 (soup, melon starter and spirits) all had a corresponding crude RR < 1.

**Table 1.** Single variable associations between food items eaten and risk of campylobacteriosis during an outbreak at a hotel in England, December 2016 (*n* = 19)

Exposure	Exposed			Unexposed			RR (95% CI)	*P*
	Total	Cases	AR	Total	Cases	AR		
Food items								
Chicken liver pâté	21	17	80.95	32	2	6.25	12.95 (3.33–50.36)	<0.001
**Soup**	**29**	**6**	**20.69**	**24**	**13**	**54.17**	**0.38 (0.17–0.85)**	**0.020**
Mints	22	11	50.00	31	8	25.81	1.94 (0.93–4.01)	0.088
Melon starter	9	1	11.11	44	18	40.91	0.27 (0.04–1.78)	0.133
Turkey	28	12	42.86	25	7	28	1.53 (0.72–3.27)	0.390
Christmas pudding	14	6	42.86	39	13	33.33	1.29 (0.61–2.72)	0.535
Crepe	3	0	0.00	50	19	38	0.00 (–)	0.545
Posset desert	17	5	29.41	36	14	38.89	0.76 (0.33–1.76)	0.555
Chocolate brownie	24	8	33.33	29	11	37.93	0.88 (0.42–1.83)	0.780
Beef	18	6	33.33	35	13	37.14	0.90 (0.41–1.96)	1.000
Cod	4	1	25.00	49	18	36.73	0.68 (0.12–3.86)	1.000
Drinks								
Spirits	11	1	9.09	42	18	42.86	0.21 (0.03–1.42)	0.074
Orange	1	1	100.00	52	18	34.62	2.89 (1.99–4.20)	0.358
Tea	1	1	100.00	52	18	34.62	2.89 (1.99–4.20)	0.358
Sparkling water	1	1	100.00	52	18	34.62	2.89 (1.99–4.20)	0.358
Diet cola	10	5	50.00	43	14	32.56	1.54 (0.72–3.27)	0.465
Coffee	12	3	25.00	41	16	39.02	0.64 (0.22–1.83)	0.502
Still water	18	7	38.89	35	12	34.29	1.13 (0.54–2.37)	0.770
Ice	4	1	25.00	49	18	36.73	0.68 (0.12–3.86)	1.000
Cola	7	2	28.57	46	17	36.96	0.77 (0.23–2.65)	1.000
Red wine	9	3	33.33	44	16	36.36	0.92 (0.34–2.50)	1.000
Beer	10	4	40.00	43	15	34.88	1.15 (0.48–2.72)	1.000
White wine	10	4	40.00	43	15	34.88	1.15 (0.48–2.72)	1.000
Apple juice	0	0	–	53	19	35.85	–	–
Cranberry juice	0	0	–	53	19	35.85	–	–

AR, attack rate (%); CI, confidence interval; RR, relative risk.

The crude association between consumption of chicken liver pâté and the risk of campylobacteriosis remained strongly statistically significant (all *P* < 0.001) in three separate sensitivity analyses (excluding probable cases (*n* = 12); excluding cases with an incubation period <24 h (*n* = 4); excluding cases with reported exposures to at least one risk factor associated with sporadic campylobacteriosis (*n* = 7)).

The significant crude associations between consumption of melon and soup and the reduced risk of campylobacteriosis were due to the choice of one starter from the set menu. A small number of individuals who ate soup also ate chicken liver pâté (5/29, 17%) and only one person who ate melon also ate chicken liver pâté (1/9, 11%). Stratified analysis demonstrated confounding by chicken liver pâté consumption for both melon (RR_M–H_ = 0.75, 95% CI 0.25–2.29) and soup (RR_M–H_ = 1.15, 95% CI 0.64–2.06).

#### Multivariable analysis

After adjustment for age group and sex, only chicken liver pâté was significantly associated with campylobacteriosis (Table 2).

**Table 2.** Multivariable model for associations with campylobacteriosis during an outbreak at a hotel in England, December 2016 (*n* = 19)

Exposures	aOR (95% CI)	*P*
Food items		
**Chicken liver pâté**	**86.57 (7.56–991.44)**	**<0.001**
Mints	7.26 (0.58–90.16)	0.123
Spirits	0.06 (0.00–2.42)	0.135
Male sex	3.20 (0.23–45.48)	0.390
Age group (years)		
0–19	1.00	–
20–39	1.96 (0.02–191.60)	0.774
40–59	1.52 (0.01–162.08)	0.861
≥60	Ref.	–

aOR, adjusted odds ratio; CI, confidence interval; Ref., reference group.

### Microbiological and environmental investigation

There was no food left from any of the meals consumed by the cases. On 5 January 2017, eight environmental swabs (equipment used for the preparation of ready to eat food (including a dedicated carving knife and cutting boards), hand touch points within the kitchen, work surfaces) and two food samples (a chicken liver pâté prepared on 31 December and a bagged leaf salad) were taken. A further sample of confit pork, which was prepared and frozen on 20 December, was taken on a subsequent follow-up visit. No food or environmental samples tested positive for *Campylobacter* spp. and all samples were deemed satisfactory for indicator organisms.

The condition of the kitchen was found to be reasonable, with most food items labelled and stored correctly. Temperatures of refrigeration equipment were good. No observations of cross-contamination were witnessed, but several issues were observed as not being consistent with good hygiene practices and indicated a potential for cross-contamination to occur.

The preparation of chicken liver pâté, as described by the chef, involved firstly cooking the chicken livers, using a probe to establish that the largest liver had reached a temperature of 75 °C. The livers were then blended with butter and eggs, passed through a sieve and confit of pork folded into the blended livers before cooking again at 100 °C for 60 min. No additional temperature checking was carried out at this stage. No written records were available to support these processes other than occasional probe checks on joints of meat.

## Discussion

Despite the existence of guidance to reduce the risk of *Campylobacter* infection associated with consumption of chicken liver containing dishes, outbreaks continue to occur. Here we have described an outbreak of campylobacteriosis strongly linked to consumption of chicken liver pâté. Despite the hotel being aware of the need to cook chicken livers properly to reduce the risk of campylobacteriosis when producing chicken liver pâté, poor record keeping could not exclude inadequate cooking during a busy period in the kitchen leading to 19 cases of acute food poisoning.

The findings of this investigation provide robust evidence for an outbreak of campylobacteriosis due to consumption of a chicken liver-containing dish. This causal relationship was supported by a clear dose–response relationship and with a strength of association robust to sensitivity analyses. No microbiological confirmation from environmental or food samples was made. Three cases reported an incubation period of <24 h, consistent with the shorter incubation periods that have been observed during other outbreaks of campylobacteriosis linked to consumption of poultry liver dishes [3]. The outbreak occurred during a busy time for the kitchen and although reported preparation practices were appropriate, no documented evidence for temperature monitoring was available. It therefore cannot be disproved with any certainty that suboptimal food preparation led to incomplete killing of *Campylobacter* during the cooking process. Although this investigation was limited to a cohort of individuals dining on a single day during the Christmas period in 2016, five other individuals with gastroenteritis reported eating at the hotel 5–7 days after the day under investigation, two of which were subsequently confirmed as having *Campylobacter* infection. As the same batch of chicken liver pâté was not likely to have been served, it seems probable that whatever ineffectual preparation methods were used on the 17 December were also applied days later. Following the outbreak, the hotel was advised by EH to stop making chicken liver pâté without being able to provide documented evidence to validate the cooking process. No further outbreaks have been reported.

Although we were unable to confirm the outbreak through microbiological sampling of food or the environment, the analytical epidemiology is robust to sensitivity in case definition, the exclusion of cases reporting a known risk factor for sporadic campylobacteriosis, and supported by a clear dose–response relationship. The exclusion of probable cases reduces the impact of case ascertainment bias on the findings while the dose–response supports a likely causal relationship between consumption of chicken liver pâté and illness. No observations of cross-contamination within the kitchen were seen, but it cannot be completely excluded that contamination of the chicken liver pâté from a secondary source had occurred in the kitchen.

Despite the availability of methods that can effectively kill *Campylobacter* during cooking [14, 15], outbreaks associated with poultry liver-containing dishes, particularly of chicken origin, continue to be reported, most likely due to inadequate cooking and preparation in food-service settings [8 13]. Outbreaks of campylobacteriosis linked to poultry liver continue to occur and a strategic communications strategy from relevant food safety and public health authorities may be required in England and elsewhere. Such a strategy should ensure that the risk profile of poultry liver-containing dishes is raised and the availability of evidence-based preventative strategies for food preparation promoted.

The outbreak we have described here highlights the need to ensure that food safety procedures are properly validated, monitored and reviewed to ensure that appropriate cooking methods are used in practice. Review of temperature and duration of cooking is especially important if any changes to standard cooking practices are made. Early identification of unsafe cooking practices can prevent future outbreaks. Nonetheless, where cooking preferences for undercooked livers remain [13 16], it seems likely that outbreaks of campylobacteriosis associated with chicken liver pâté served at catered events and restaurants will continue to occur.
